# Optimising Human Mesenchymal Stem Cell Numbers for Clinical Application: A Literature Review

**DOI:** 10.1155/2012/465259

**Published:** 2012-01-31

**Authors:** E. Fossett, W. S. Khan

**Affiliations:** University College London Institute for Orthopaedics and Musculoskeletal Sciences, Royal National Orthopaedic Hospital, Stanmore HA7 4LP, UK

## Abstract

Adult mesenchymal stem cells (MSCs) are being investigated further for their use in stem cell therapies. However, as they are found in very low numbers in adult tissue, expansion *in vitro* is required to produce desired MSC numbers for clinical application. The need for effective cell-based therapies is increasing due to a rise in the ageing population, increasing the prevalence of musculoskeletal disorders. This review investigates how factors, age and gender of donor, as well as seeding density can affect MSC expansion. Age and gender of donor have received mixed results from studies, whereas seeding density studies have produced consistent results for numerous MSC sources, favouring lower seeding densities. Further research is required to reduce the risk of infection, loss of cell characterisation in cell culture, and making cell-based therapies more cost effective through creating rapid expansion of MSCs regardless of patient factors.

## 1. Introduction

Stem cells are an undifferentiated population, capable of endless self-renewal and differentiation down one or more lineages to produce specialised cell types [1]. Their ability to produce many cell types *in vitro* is one of the characteristics that has highlighted their importance for use in cell-based therapies. The earliest stem cell in the human body, the fertilised egg, is totipotent and has the capacity to differentiate into all cell types of the human body, as well as tissues to support the embryo. As the fertilised egg develops into cells of the human embryo, differentiation capacity down lineages become more limited [2].

Adult stem cells, also known as somatic stem cells, are located in many tissues of the human body and are required to restore normal function via repair and regeneration of tissues *in vivo*, for example, satellite cells in muscle tissue. They exist in a quiescent state until activated by mediators of injury or disease. Adult stem cells make up a small percentage of cells in a tissue and are surrounded by mature cells that have reached the end of the differentiation process and do not have the capacity to proliferate or differentiate [3]. The use of adult stem cells in clinical application is being investigated further due to restrictions and ethical issues surrounding the use of embryonic stem cells. Adult mesenchymal stems cells (MSCs) are suitable for use in clinical application as they have been found to adhere well to plastic, proliferate and differentiate well *in vitro*, and have suitable properties for transplantation: low immunogenic and high immunosuppressive properties, due to a low or absent HLA-2 marker on their cell surface [4, 5]. They have currently been shown to have the potential to enhance treatment of cardiovascular [6], neurological [7], and musculoskeletal disorders [8] by differentiating into cardiomyocytes and vascular cells, neuron and glial-like cells, and chondrocytes, respectively.

An early study by Friedenstein et al. reported that MSCs isolated from bone marrow, were found to be similar to fibroblasts [4, 9, 10]. These cells were clonogenic, adhered to plastic in culture, and replicated extensively *in vitro*. It has been demonstrated that these cells are multipotent, differentiating into osteoblasts, chondrocytes and adipocytes, when transplanted back *in vivo* [11, 12]. As well as the isolation of MSCs from bone marrow, other sources including adipose [13], skeletal muscle [14], synovium [15] and synovial fat pad [16] have also been reported to contain MSCs capable of multilineage differentiation. 

MSCs have shown great capabilities for use in clinical application; however, as they are found in very low numbers in adult tissue, expansion *in vitro *is required to reach the desired numbers before their use in clinical application. We have yet to develop a clear understanding of how to optimise MSC expansion efficiently, as many papers have reported discrepancies in results such as how age of donor, gender of donor, and seeding density can affect cell proliferation rate. Factors affecting optimisation of MSCs can be grouped into patient factors such as age and gender, as well as cell culturing factors such as seeding density used. This review article will look at how age, gender and seeding density affects the proliferation of adult mesenchymal stem cells. 

## 2. Donor Age and MSC Proliferation

Mixed results have been published in existing literature about how age of donor affects mesenchymal stem cell expansion rate. Many have reported an inversely proportional relationship between proliferation rate and age; however, others have reported no relationship. Most literature has used BMSCs when looking at the effects of age, with a couple of papers using infrapatellar fat- pad-derived stem cells. 

Khan et al. investigated the proliferation rate of synovial fat pad MSCs in two groups of patients with a mean age of 57 and 86, finding no significant difference in cell proliferation between both groups at five time points (days 2, 4, 6, 8, 10) [17]. This trend has also been supported by many papers investigating the effect of age using bone marrow-derived mesenchymal stem cells (BMSCs). Phinney et al. found up to 12-fold differences between patients when investigating growth properties of BMSCs from 17 healthy patients aged 19–45 years old; however, this difference showed no correlation with age of donor (*P* < 0.05) [18]. Both Suva et al. and Scharstuhl et al. extracted BMSCs from the neck and shaft of femur, respectively, at the time of hip arthroplasty [19, 20]. Suva et al. reported variable results for time required to reach the first passage, exponential cell growth, doubling time, and maximal cell amplification, but again none of these variations were found to be due to age-related differences of donors. Similarly with a sample size of 98, Scharstuhl et al. also reported that proliferative capacity is maintained with ageing after correlating proliferation with age. 

On the other hand, Baxter et al. reported a severely reduced proliferative capacity with slower growth rate in a group of 59–75 years old patients compared to 0–18 years old [21]. This was supported by other studies that found that doubling time was almost 2-fold longer in older patients compared to younger [22, 23]. Culturing BMSCs over 4 months from “young” (7–18 years old), “adult” (19–40 years old), and “aged” (>40 years old) patients extracted from the posterior iliac crest, Stolzing et al. found differences between proliferation rate from week 5 in culture, describing that the proliferation rate of the “aged” BMSCs began to decrease and the growth curve started to plateau [24]. On the other hand, BMSCs from “adult” patients continued to increase in proliferation rate throughout the whole 4 months in culture. The “young” group of BMSCs were only investigated over 10 population doublings, where they also displayed a pattern of increasing proliferation rate with time. These findings were also supported by Dexheimer et al., who found a significant age-related decline in proliferation rate in BMSCs from older compared to younger patients [25]. Clonal expandability also decreased with increasing age with cells from an 80-year-old patient producing half the number of clones of that of BMSCs from a 20-year-old. 

Interestingly, whilst investigating the effect of age on MSC proliferation from synovial fat pad tissue, one study explored this relationship at eight different seeding densities: 50, 250, 500, 1000, 2500, 5000, 750, and 10000 cells/cm^2^ [26]. Extremely varied results were found, with five seeding densities (50, 250, 500, 5000, 7500 cells/cm^2^) showing that there was an age-related decline in population doublings, whereas 10,000 cells/cm^2^ showed an age-related increase in population doublings and two densities (2500 and 1000 cells/cm^2^), showed no correlation with age. Varied results even between the same set of cells at different seeding densities shows that properties of MSCs and how they are altered are not properly understood. It also shows that proliferation can be affected by many factors, in which more research needs to go into providing knowledge about how MSCs are affected by a range of factors. 

It is not known whether ageing of MSCs is due to factors inside the cells or factors in the surrounding tissue. It has been found that reduced synthesis of proteoglycans and glycosaminoglycans in the surrounding tissue results in a reduced proliferation and viability of MSCs *in vivo* [27]. Also, the production of advanced glycosylated end products (AGEs) inhibit proliferation of MSCs by activating apoptosis and reactive oxygen species (ROS) production [28]. In comparison, Zhou et al. suggested that intrinsic factors may be responsible for age-related changes in BMSCs [22]. He found that there was a 4-fold increase in the number of MSCs that were positive for senescence-associated *β*-galactosidase (SA- *β*-gal) and increased expression of p53 and its pathways genes (p21 and BAX) that may be responsible for mediating reduced proliferation potential. It has also been suggested that the expression of p16INK4a, an inhibitor of CDK4 and CDK6 which promote proliferation, increases with age [29]. MSCs from older patients were also found to be more apoptotic as physiological effects of ageing on MSCs induce senescence [22]. 

The effect of ageing on the properties of MSC remains controversial with inconsistent results, even when using MSCs from the same source type and patient. It is important to investigate how age affects the proliferation rate of mesenchymal stem cells to provide knowledge as to whether stem cell therapies can be achieved via autologous repair for older patients. This is a question which is becoming of great importance as with an ageing population, the prevalence of musculoskeletal disorders is increasing. Some studies have also shown that a higher proliferation rate is related to stronger osteogenic, chondrogenic, and adipogenic differentiation potential, crucial for repair of damaged or diseased musculoskeletal tissue [25]. The variable results could be due to studies having a restricted age range due to the patient cohort available, studies having different age ranges for “young” and “old” categories, or not having a large enough sample size for young patients as quite a few studies use BMSCs extracted during hip arthroplasties, which on average occur more frequently in the older population. The variable results between studies call for more work on this subject to be undertaken to get a clear idea of how age of donor affects MSC proliferation rate. In particular, different sources of MSCs should be investigated and perhaps, if ageing is found to affect MSC expansion, then one source may be less affected and more suited to cell based therapies for the older population. 

## 3. Gender and MSC Proliferation

There is little literature on the effects of gender on MSC proliferation potential, as the majority of papers group results from male and female patients together. MSC culturing techniques, such as duration and concentration of collagenase, and best harvesting site for maximal yield vary between males and females [30, 31]. Faustini et al. using adipose-derived stem cells (ADSCs) discovered that ADSC underwent most effective digestion when adipose tissue was incubated with 0.2% collagenase for 1 hour for males, whereas overnight digestion was more effective for tissue from females [31]. Females having a significantly higher yield of MSCs than males were also reported, with females having on average 2.05 ± 1.46 log-million cells compared to 1.44 ± 1.62 log-million cells from males. This, along with other knowledge that stem cells have estrogen and androgen receptors, suggests that there can be gender differences in regards to MSC proliferation. The higher levels of estrogens in females and androgens in males are thought to be responsible for the differences in male and female MSCs [30]. Most studies suggest that androgens have an inhibitory effect on the function of stem cells [32], whereas estrogens have an excitatory role controlling levels of cytokines and growth factor production in MSCs [33]. Estrogens have also been found to upregulate the expression of receptors on embryonic stem cells (ERa and ERb), increasing the production of 17b- estradiol which activates MAPK and cyclin-dependant kinases (cdk), which are intermediates in the cell cycle [34]. 

Despite suggestions and findings in some studies, Dexheimer et al. found no gender differences in MSC frequency and expansion of nonclonal MSC populations from bone marrow [25]. As gender could be a factor of influence for reaching desired cell numbers, research investigating the effect of gender on the properties of MSCs needs to be undertaken. This will help to make extraction and expansion of MSCs more efficient for all patients undergoing cell-based therapies as it can be known to extract more tissue from males than women, if females do have a higher yield as Faustini et al. reported. Also, more work should investigate the preferred isolation and culturing techniques for MSCs from male and female patients to ensure that the maximum yield of MSCs is extracted from the tissue source as possible.

## 4. Seeding Density and MSC Proliferation

Although limited, the literature suggests that seeding density does have an effect on cell proliferation rate. Both et al. found that BMSCs seeded at lower densities had a faster proliferation than higher densities, with MSCs at 100 cells/cm^2^ reaching their target of 200 million cells, 4.1 days faster than cells that were seeded at a higher density (5000 cells/cm^2^) [35]. Further decrease in seeding density below 100 cell/cm^2^, also showed a further increase in proliferation rate. A similar relationship was found by Lode et al., when investigating the effect of seeding density on three-dimensional scaffolds [36]. They found that the highest seeding density of 1 × 10^6^ cell/cm^2^ resulted in a minimal increase in cell number compared to the lowest seeding density, which had a large increase in cell number. Extremely low densities (0.5–12 cells/cm^2^) showed the size of single-cell-derived colonies that represent cell number and hence proliferation rate, to be inversely proportional to seeding density [37]. Another study looking at four different seeding densities for BMSCs also had results that were consistent with the previous findings. After 10 days in culture, BMSCs seeded at 2500, 250, 25, and 2.5 cells/cm^2^ had mean results of 2.72 ± 0.48, 4.8 ± 0.42, 6.7 ± 0.53, and 7.6 ± 0.97 population doublings, showing that cells at a lower density have a faster proliferation rate than those of a higher density. Significant gain in population doublings was seen between 2500 and 250, as well as 250 and 25 (*P* < 0.05). On the other hand, although more population doublings occurred at lower densities, there was no significant difference between using 25 and 2.5 cell/cm^2^. They also showed that seeding density did not affect cell characterisation as all cells had the same cell surface marker characterisation profiles [38]. This trend has been shown to occur in MSCs from various tissue locations not only bone marrow, confirming that seeding density does have a significant effect on proliferation rate. A study was carried out by Mochizuki et al. exploring the optimum seeding density for bone marrow-, synovium-, periosteum-, adipose-, and skeletal muscle-derived mesenchymal stem cells. Measuring cell growth and colony formation using crystal violet staining, they found that all MSCs had a greater colony size at lower seeding densities compared to higher; for example, MSCs derived from synovial tissue showed smaller colony size or cells became indistinct after 14 days when seeded at 10^5^ and 10^6^ cells/60 cm^2^, compared to seeding densities of 10^3^ or 10^4^ cells/60 cm^2^. Results showed that all sources had an optimum seeding density of 10^3^ or 10^4^ cells/60 cm^2^or 10^3^/10^4^ cells per cm^2^ for BMSCs. In addition similar trends were seen when cells were seeded at 50 cells/cm^2^ producing a higher cumulative number of cells than at 5,000 cells/cm^2^. They also suggested that seeding density and total cell doubling times can affect senescence of cells. Synovial fat-pad derived MSCs observed results that were consistent with those from BMSCs [39].

The lower growth rate of cells seeded at higher densities could be due to contact inhibition. Higher growth rates at lower densities have been explained by the presence of small and agranular cells, also referred to as recycling stem cells, in the lag phase, which gave rise to large cells during the log phase of exponential growth [37]. The log phase has been found to last for a longer duration in cells seeded at lower densities, and hence more population doublings occur due to a longer exponential growth phase [40]. Higher growth potential at lower seeding densities may also be due to more availability of nutrients per cell.

Finding the optimum seeding density for maximal expansion is useful in both laboratory investigations as well as potential clinical applications as the cell culturing procedure can be less time consuming, decreasing the risk of cell culture contamination, infection or loss of characteristics in cell culture, in addition to making the process more cost effective. These studies show that rapid expansion to reach a sufficient number of cells for clinical applications can be achieved by using lower seeding densities. As numerous papers have found that MSCs from a range of sources all have a faster proliferation rate/population doublings at lower seeding densities, if consistent relationships are found between age of donor and proliferation rate or gender and proliferation rate, then perhaps seeding density can be used to compensate for and be used to speed up expansion of MSCs for clinical application. 

This review has looked at how some factors such as age and gender of donor as well as seeding density can affect MSC expansion for clinical application. Whilst studies disagree on the effects of ageing on MSCs, more work should be carried out to explore the effect of age on numerous MSC sources in the hope that a consistent relationship can be found. Identification of relationships will enable cell culturing techniques to be adapted for expansion of MSCs from particular age groups of patients, without increased risk of infection in cell culture by minimising time spent in culture. Gender is also a factor that should be investigated fully, as exposure to different concentrations of hormones may alter properties of MSCs. Research of how age and gender affects MSC proliferation should make sure that apart from the factor being investigated patients are all matched by gender, age, social factors, medical history, and chronic illness to ensure that all results are not due to potential confounders. In comparison, seeding density has shown consistent results over numerous studies as well as for numerous MSC sources. As the relationship between seeding density and proliferation rate of MSCs has been found, once age and gender relationships are also investigated, seeding density could perhaps be used to compensate for the effect of patient factors to result in rapid expansion of MSCs. 
